# A validation of the Swedish self-concept and Identity Measure (SCIM) and its association with mental health problems

**DOI:** 10.1016/j.heliyon.2023.e18151

**Published:** 2023-07-11

**Authors:** Rosie James, Daiva Daukantaité, Magnus Nilsson

**Affiliations:** a Faculty of Medicine at Lund University, Sweden; b Department of Psychology, Lund University, Sweden; c Department of Clinical Sciences Lund, Psychiatry, Lund University, Sweden; d Clinical Psychiatric Research Centre, Region Skåne, Lund, Sweden

**Keywords:** Clinical identity disturbance, Confirmatory factor analysis, Depression, Anxiety, Borderline personality disorder, Non-suicidal self-injury, Validation

## Abstract

Pathological disturbance to one's identity is closely linked with mental illness and in particular personality disorders. Current measures of identity pathology within clinical research are nevertheless inconsistently used and present with substantial limitations such as disproportionate focus on adolescence. The Self-Concept and Identity Measure (SCIM) identifies pathological and non-pathological identity disturbance by implementing a measurement for clinical components of identity, as well as introducing the Lack of Identity concept. This study thus explores the psychometric properties (factor structure, internal consistency, and criterion validity) of the Swedish SCIM in a large sample of Swedish university students (N = 1500). Model fit indices for the three-dimension model of identity pathology consisting of consolidated-, disturbed-, and lack of identity subscales were deemed acceptable and the Swedish SCIM scores correlated with measures of psychopathology in the expected direction, together concluding that the Swedish SCIM was satisfactorily valid and reliable. The results further reveal a significant positive correlation between identity pathology and non-suicidal self-injury, two concepts that co-occur in psychopathologies, such as borderline personality disorder, but have not yet been studied in a Western population with this tool. The potential clinical use of this translated dimensional tool needs to be tested in a Swedish clinical population, however, we conclude that it already offers insight into the complexities of identity functioning and correlations with clinical symptoms.

## Highlights

• Identity pathology is a fundamental symptom of personality disorders.
• We are lacking measures for normal and pathological identity problems in adults.
• The paper investigates the Self-Concept and Identity Measure (SCIM) in Swedish.
• Results suggest that the SCIM is a reliable and valid tool in Swedish populations.
• We highlight the clinical significance of the “Lack of Identity” subscale.

## 1 Introduction

Identity is a sense of self-continuity, and the subjective experience of knowing who we are and our place in society [1]. Early work describes this normal developmental process in adolescence as questioning and then committing to an “adult” identity [2,3]. This typical identity development is highly studied in adolescents [[4], [5], [6]], however, little work is done on adult identity pathology from a clinical perspective [7,8]. The need for clinical research taking these into account is emphasised by the theoretical framework linking identity problems with psychopathology as proposed by Klimstra and Denissen in 2017. They highlight the complexity of identity pathology with regard to, among other things, the many dimensions of individual identity. As of now there is no validated Swedish self-report measurement of identity pathology in adults. As identity pathology and non-suicidal self-injury (NSSI) are characteristic of borderline personality disorder (BPD), which is drastically increasing in Sweden [9], a validation of the translated SCIM is needed to empirically explore this understudied symptom of a common disorder, especially its relation to NSSI.

### 1.1 Measuring identity pathology

The latest diagnostic and statistics manual (DSM-5-TR) recommends the assessment of identity pathology severity when diagnosing personality disorders (PDs) as part of the Alternative Model to Personality Disorders (AMPD) [10]. Although not the principal model for diagnosis, its inclusion follows the strong evidence of a dimensional view of psychopathology and the importance of identity pathology when diagnosing PDs [[11], [12], [13], [14]]. Identity pathology is thus emphasised as a central symptom of PDs; however, a recent review suggests that clinical literature still deprioritises PD severity (that take identity problems into account) in participants in favour of categorically assessing if the person has a PD or not based on observed symptoms [13,15]. Likely reasons for this lie in the difficulty of measuring the internal processes involved in identity and the simplicity of categorisation [16,17]. This trend is however at the expense of contributing to research on a complete picture of PDs with dimensional and severity aspects of identity problems considered, as well as being an oversimplification of a diagnosis that already suffers from high heterogeneity [17].

To encourage the investigation of dimensions and severity in clinical research, clinically relevant measurement scales that incorporate these concepts are needed. Current measures of identity disturbance however focus primarily on adolescents [8] and usually assign identity-related categories as opposed to severity levels [2]. Not only does the focus on adolescents exclude a substantial part of the population diagnosed with PDs, as the majority are diagnosed only after reaching adulthood [15], but by assigning categories within identity research we lose the complexity of identity dimensions and severity of overall functioning (Klimstra & Denissen, 2017).

In 2015, Kaufman and colleagues aimed to overcome these limitations by developing the Self-Concept and Identity Measure (SCIM), a self-report questionnaire operationalising normal and pathological identity problems in adults.

### 1.2 The Self-Concept and Identity Measure (SCIM)

Incorporating traditional identity research by Erikson with an extensive review of more recent literature and clinical accounts of identity pathology, the SCIM was formed with three dimensional subscales: Consolidated-, Disturbed-, and Lack of Identity [18]. Consolidated Identity encompasses a measure of healthy identity functioning, which closely links to Erikson's descriptions of a successful identity development process in adolescence [3,18]. Disturbed Identity refers to problems such as Erikson's *identity confusion*; an uncertain and diffuse grip on one's role and purpose in the world [18]. Lack of Identity is a subscale unique to the SCIM within which the authors aimed to capture severe identity distress seen in those with psychopathological diagnoses. Clinical descriptions of borderline personality disorder (BPD) were a primary influence, due to the feeling of lacking an identity altogether having a reoccurring yet unexplored presence in BPD patients [18].

Since the original validation on a non-clinical, English-speaking sample [18], Kaufman and colleagues have additionally demonstrated the SCIM's usefulness within a treatment-seeking sample of those with substance abuse and comorbid mental health problems such as depression and emotion dysregulation [19]. Post-creation adjustments to the English scale have not been motivated despite validation results not being ideal, however the Dutch validation resulted in an adjustment to the scale [20]. These three papers additionally demonstrated good construct and criterion validity through expected correlations with depression, anxiety, BPD, and emotion dysregulation. An Indian translation of the SCIM has been used recently to identify the link between NSSI and identity functioning, however the translation has not been validated as of yet [21]. However, considering the relationship between NSSI and PDs [22,23] as well as NSSI's link with identity-related problems in adolescence [24,25] it is surprising that NSSI has not yet been tested with respect to the SCIM in the Western context.

### 1.3 Present study aims

To evaluate the psychometric properties (factor structure, internal consistency, and criterion validity) of the Swedish translated SCIM.

To explore the SCIM's relationship with self-reported depression, anxiety, BPD symptoms, and NSSI, as a measure of criterion validity.

## 2 Method

### 2.1 Procedure

A comprehensive survey was sent out to 32,001 student email addresses in February 2022 which resulted in convenience sample of 1500 responses (response rate of 4.7%). All participants were over the age of 18 and gave informed consent before completing the survey. The data was obtained from the list of those registered for courses at Lund University between September 2021 and January 2022. The response rate is not fully representative however since it may have been possible that some students discontinued their attendance of the courses and the email addresses were inactive, alternatively that emails were filtered out and not seen. All confidential data was stored at the approved data-storage service used by the Medical Faculty at Lund University, LUSEC. Participation was voluntary, anonymous and the study was approved by the Swedish Ethical Review Authority (Dnr 2021–05102). No compensation was offered to participants, however proven psychological shortcut strategies were used in which participants were more likely to respond as the sender was the university, seen as a legitimate authority, and a follow up email after 14 days was sent to those who did not initially respond [26].

### 2.2 Measures

#### 2.2.1 Self-concept and Identity Measure

The SCIM consists of 27 statements rated on a 7-point Likert scale from 1 (*Completely disagree*) to 7 (*Completely agree*). Examples of statements in the three subscales are: “I always have a good sense about what is important to me” for Consolidated Identity, “I imitate other people instead of being myself” for Disturbed Identity and “I am broken” for Lack of Identity. Total SCIM score was calculated by reverse coding items in the Consolidated Identity subscale and then summing the three subscales. Evidence suggests that the SCIM is reliable and structurally valid among both non-clinical (α = 0.89) and clinical populations (α = 0.86) [18,19].

#### 2.2.2 Depression and anxiety

Depression symptoms were assessed using the Swedish Symptom Checklist-core depression (SCL-CD6; Magnusson Hanson et al., 2014), a brief six-item scale looking into how much people have been bothered by things such as “feeling blue/sad” and “blaming yourself”. It uses a five-point Likert scale from 1 (*Not at all*) to 5 (*Extremely*), which has excellent internal reliability where Cronbach's α has been 0.92 [27]. In the study, Cronbach's α was 0.90.

Anxiety symptoms were assessed using the Swedish Generalised Anxiety Disorder (GAD-7; Spitzer et al., 2006) questionnaire, consisting of seven questions about how participants have been feeling during the last two weeks using a four-point Likert scale from 0 (*Not at all*) to 3 (*Daily*). Previous research using the Swedish GAD-7 has shown good internal consistency with a Cronbach's α of 0.92 [28] and Cronbach's α was 0.91 in the present study.

#### 2.2.3 Emotion regulation, BPD and NSSI

The Brief Version of the Difficulties in Emotion Regulation Scale (DERS-16; Bjureberg et al., 2016) included 16 statements such as “I have difficulty making sense out of my feelings” and how often they applied to participants on a five-point Likert scale from 1 (*Almost never*) to 5 (*Almost always*). Internal consistency for the Swedish DERS-16 has previously been very high with Cronbach's α between 0.92 and 0.94 [29]. Cronbach's α was 0.93 in this study.

BPD symptoms were assessed using the McLean Screening Instrument for BPD (MSI-BPD) [30] where participants answer either yes or no to ten questions, for example “Have you chronically felt empty?“. It has been translated and validated in many languages, showing good internal consistency [31], although not validated in its Swedish translation its successful use in published research on Swedish populations means that it can offer insight into the measure [32,33]. Our research shows a Cronbach's α of 0.77.

To measure NSSI, participants were asked about their history of self-harm using the definition of intentionally and repeatedly hurting oneself without the aim to die e.g., by cutting, scratching, poking, burning, or hitting yourself [34]. There were four possible answers: “earlier in life but not during the last six months”, “within the last six months but not before”, “both within the last six months and earlier”, and “neither within the last six months nor before”. For the purposes of this study, these were divided into two categories, 0 (*those who had never self-harmed*) and 1 (*those who had self-harmed at some point*).

### 2.3 Data analyses

**Fig. 1:**
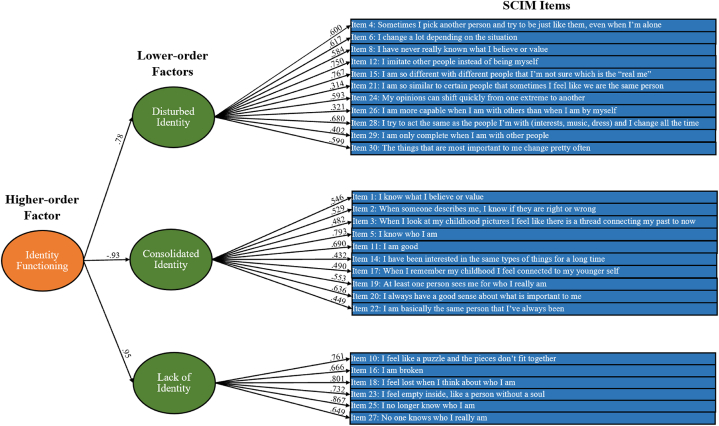
SCIM confirmatory factor analysis model with factor loadings all significant to *p* < .001.

Confirmatory Factor Analysis (CFA) using the AMOS addon to SPSS [35], was run with a maximum likelihood estimator. Like the authors of the original scale, we used items 16, 12 and 20 (see Fig. 1 For more information) as theoretical anchors due to their respective representativeness of the Disturbed-, Consolidated-, and Lack of Identity subscales and constrained the other items to load onto each factor according to the proposed three-factor model [18]. Lack of Identity worked as the anchor for the higher-order, Identity Functioning factor. To assess if the established model of SCIM fit the translated SCIM data, the root mean square of approximation (RMSEA) with 95% confidence intervals, the comparative fit index (CFI), and the standardized root mean residual (SRMR) were used as criteria. An acceptable model fit was defined as CFI ≥0.90, RMSEA ≤0.08, and SRMR ≤0.10 [36,37].

The SCIM was subsequently tested for measurement invariance between women and men [38,39]. For model comparison, we relied on CFI, RMSEA and SRMR as has become common practice [40], due to the sensitivity of chi-square difference tests to sample size [41,42]. Therefore, we considered values of ΔCFI<0.01, ΔRMSEA<0.015, and ΔSRMR<0.03 as indicating sufficient model similarity to conclude metric and scalar invariance [41,42].

Cronbach's α values of above 0.70 was deemed adequate for internal consistency. Criterion validity was measured in terms of Pearson's correlation coefficient *r* of SCIM scores and psychopathology scores. This was interpreted according to Cohen's guidelines of > 0.10 as small, >0.30 as medium, and >0.50 as large [43].

### 2.4 Translation procedure

The original, English SCIM [18] was translated into Swedish using the ‘back-to-back’ method [44], whereby author MN (PhD and clinical psychologist with research and clinical experience in BPD), with the support from Lars-Gunnar Lundh, professor in clinical psychology, created the Swedish translation, this was then translated back into English by Reid Lantto, a native English speaking clinical psychologist and researcher (extensive experience of BPD research and practice) who was unaware of the original scale. The translated version was checked and agreed on by authors MN and DD (associate professor and researcher in clinical psychology) as well as another clinical psychologist, Elin Lundgren, via email correspondence. The consensus was that no items would be removed and that no more adjustments were needed after this translation process.

## 3 Results

### 3.1 Respondent characteristics

1500 students responded with an average age of 26.37 years (SD = 7.57) attending Lund University (LU), Sweden. See Table 1 for details on participant characteristics.

**Table 1:** Respondent characteristics.

Characteristic	Total n = 1500
Age, years, mean (SD)	26.37 (7.57)
Gender identity, *n* (%)	
Female	1024 (68.3)
Male	424 (28.3)
Other	38 (2.5)
Prefer not to say	14 (0.9)
Current living situation, *n* (%)	
With a partner or spouse	412 (27.5)
Alone	537 (35.8)
With parents (or childhood family)	191 (12.7)
With other adults (e.g., student housing)	320 (31.3)
Other	40 (2.7)
Parentage, *n* (%)	
Both parents born in Sweden	1099 (73.3)
One parent born in Sweden	202 (13.5)
Both parents born outside Sweden	199 (13.3)
Place of birth, *n* (%)	
Born in Sweden	1297 (86.5)
Born in a Nordic country^1^	39 (2.6)
Born outside a Nordic country	160 (10.7)
Relationship, *n* (%)	
Single	798 (53.2)
Boyfriend/girlfriend	543 (36.2)
Married/partnership	159 (10.6)

*Notes.*^1^Nordic countries include Norway, Denmark, Finland and Iceland.

### 3.2 Confirmatory factor analysis and measurement invariance

All items loaded significantly (*p* < .001) onto the three lower-order factors taken from the subscales (Disturbed-, Consolidated-, and Lack of Identity) that also loaded significantly onto the higher-order Identity Functioning factor (see Fig. 1). Results showed the following model fit (χ^2^ (321) = 3414.72, p < .001; CFI = 0.82, TLI = 0.81, RMSEA = 0.08 [CI = 0.078, 0.083], SRMR = 0.06). Although SRMR and RMSEA showed adequate model fit, CFI did not reach an adequate fit unless the error residuals of six pairs of items were correlated (items 3 and 17, 4 and 12, 16 and 23, 1 and 20, 10 and 16, and 30 and 24, the pairs are from the same subscales) and five items removed (items 14, 21, 22, 26 and 29, which had low (i.e., below 0.40) loadings), as suggested by modification indices. The adjusted model showed the following fit (χ^2^ (200) = 1722.81, p < .001; CFI = 0.90, TLI = 0.89, RMSEA = 0.071 [CI = 0.068, 0.074], SRMR = 0.496). However, CFI did not increase enough to warrant a model adjustment with so many restrictions in line with previous reasonings [18,19].

Cronbach's α for the total SCIM score and all subscales were excellent and are shown with means and standard deviations in Table 2. Scalar invariance indicated that mean values are directly comparable between women and men (see Table 3).

**Table 2:** Self-concept and Identity Scale descriptives.

Measurement	Mean	SD	α
SCIM Total	69.40	22.94	.92
Disturbed Identity subscale	28.78	10.36	.84
Consolidated Identity subscale	51.34	9.41	.81
Lack of Identity subscale	16.62	8.38	.88

**Table 3:** Comparing configural, metric and scalar invariance between women and men.

Invariance	df	AIC	BIC	χ2	Δχ2	p	CFI	RMSEA	SRMR	ΔCFI	ΔRMSEA	ΔSRMR
Configural	642	131198.82	132085.52	3811.63			.816	.083	.060			
Metric	666	131194.57	131954.60	3855.38	43.75^1^	.008	.814	.081	.063	.002	.002	.003
Scalar	690	131338.60	131971.95	4047.41	192.02^2^	<.001	.805	.081	.065	.009	.000	.002

*Note.*^1^Metric against Configural; ^2^Scalar against Metric; AIC = Akaike information criterion; BIC = Bayesian information criterion; CFI = comparative fit index; TLI = Tucker–Lewis index; RMSEA = root mean square error of approximation; SRMR = standardized root mean square residual.

### 3.3 Associations with measures of psychopathology

Using bivariate correlation analyses the relationships between total SCIM scores and measures of anxiety, depression, BPD, and DER were significant and moderately positive (see Table 4). Dummy variables were used for the two NSSI groups (as described in 2.2.3.) and results showed a statistically significant, medium-sized correlation between having at some point self-harmed and having a higher total SCIM score.

**Table 4:** Pearson's correlations between psychopathology variables and the SCIM with 95% confidence intervals.

Variable	1.	2.	3.	4.	5.	6.	7.	8.
1. Depression	–							
2. Anxiety	.79 (.76–.82)	–						
3. Emotion dysregulation	.70 (.67–.74)	.68 (.64–.71)	–					
4. BPD	.61 (.57–.65)	.59 (.55–.63)	.68 (.64–.72)	–				
5. NSSI	.29 (.24–.34)	.32 (.27–.37)	.39 (.34–.44)	.38 (.33–.43)	–			
6. SCIM total	.56 (.51–.60)	.50 (.45–.54)	.62 (.58–.66)	.59 (.55–.63)	.30 (.25–.35)	–		
7. Disturbed Identity	.39 (.35–.44)	.36 (.31–.41)	.48 (.43–.52)	.47 (.42–.51)	.19 (.14–.24)	.86 (.83–.88)	–	
8. Consolidated Identity	−.48 (−.52 to −.43)	−.41 (−.46 to −.37)	−.50 (−.54 to −.46)	−.46 (−.51 to −.42)	−.28 -.33 to −.23)	−.83 (−.86 to −.80)	−.57 (−.61 to −.53)	–
9. Lack of Identity	.65 (.61–.69)	.57 (.53–.61)	.68 (.64–.71)	.65 (.61–.69)	.34 (.29–.38)	.87 (.84–.89)	.62 (.58–.66)	−.72 (−.76 to −.69)

*Note*. N = 1500. SCIM Consolidated Identity subscale reversed for SCIM total score but not in the individual sub-score. Dummy variable used for NSSI where 1 is having self-harmed at some point and 0 is never having self-harmed; all correlations are statistically significant to *p* < .001 and reported in Pearson's *r.* 95% confidence intervals are reported in brackets following each correlation coefficient.

Each subscale was significantly correlated with the psychopathology measures in the expected directions with at least medium strength, apart from NSSI which showed only a small correlation with the Disturbed Identity subscale (see also Table 4). The Lack of Identity subscale showed the strongest significant associations with all measures, ranging from *r* = 0.335 with NSSI to *r* = 0.676 with DER.

## 4 Discussion

The present study examined the psychometric properties (factor structure, internal consistency, and criterion validity) of the Swedish Self-Concept and Identity Measure (SCIM) in a large sample of Swedish university students. The study additionally evaluated the associations between the SCIM subscales and mental health problems including NSSI, depression, anxiety, and BPD symptoms.

The factor analysis indicated generally good construct validity for the Swedish SCIM's three-dimensional model of identity functioning: Consolidated Identity, Disturbed Identity and Lack of Identity. Results replicate the original model which has since also been validated among English and Dutch speaking clinical and non-clinical populations [[18], [19], [20],^45^]. Similarly to other validations some items that had lower loadings led to a less than ideal fit that different authors handled in various ways. However due to these lower loading items being different from those identified in other versions, we decided not to adjust the model in favour of parsimony. The fact that the lowest loadings differed from other translations could imply some cross-cultural nuances that are difficult to control for when translating a scale, but not necessarily a problem with the item constructs themselves. This was shown by the strong criterion validity achieved by the Swedish SCIM and its subscales due to expected relationships with psychopathologies similarly reported by other validations of the SCIM [[18], [19], [20],^45^].

Of note, was the significant correlation between NSSI and pathological identity as shown by the total SCIM score, a link recently identified in a non-Western population [21]. Our results differ however due to the significant positive correlation between NSSI and the Lack of Identity dimension, which was not previously identified. The difference could be explained by the concept of identity not being directly comparable between Western and non-Western cultures, especially when using a measure developed and validated within Western populations [46]. Our finding may be particularly clinically relevant in the context of Western psychiatry, where a focus on individuality and feeling of “lacking” an identity could be more prevalent [46]. The addition of this third dimension within the broader view of identity functioning enables the measurement of a less recognised but equally important element as a part of identity functioning. Clinical research can benefit from the exploration of identity pathology from this lesser known three-factor perspective, and clinical practice could benefit from a scale that investigates a more nuanced, dimensional understanding of PD pathology which reflects the more recent advances in PD research [13,17,47].

### 4.1 Further research

Although the current model fits this study's data well enough, it would be important to see whether similar fit indices appear in a clinical sample in Sweden, given that identity disturbance is recognised as one of the symptoms of BPD. Since the low loading items in this study differed from those in other validations, it could be necessary to revise the scale using different international samples aiming for cross-cultural applicability. It would additionally be worth seeing if the SCIM could be adapted to the clinical setting. Lastly, we suggest further exploration of the interaction between NSSI and identity pathology specifically looking at the concept of lacking an identity.

### 4.2 Strengths and limitations

Strengths include the large sample size of 1500 participants despite the 5% response rate (a conservative calculation). The slight overrepresentation of women reflects the greater proportion of women studying at Lund university at the time [48]. Methodological limitations include not obtaining a level of agreement for the translated scale and not using a measure of BPD symptoms validated in Swedish. There is also a response bias lowering the generalisability of the study, firstly participants were all students and secondly there could be a difference between those who voluntarily answered and those who declined. Lastly there is a lack of controlled conditions in online questionnaires which could be improved with a different design, however it would likely be at the expense of the large sample size.

## 5 Conclusion

This study demonstrated sound psychometric properties of the SCIM in a Swedish student sample, showing promise for the SCIM as a tool for assessing self-reported adult identity pathology in research settings. Although more research is needed to test its use within Swedish clinical samples, this validation has provided evidence towards its future clinical relevance. Research on identity pathology, in particular the Lack of Identity concept, is still sparse despite its significance in the field of personality disorders. Thus, the validation of such a measure which considers not only developmental identity problems, but also clinically relevant, adult pathological identity issues, is important.

## Author contribution statement

Rosie James: Performed the experiments; Analyzed and interpreted the data; Wrote the paper.

Daiva Daukantaité: Performed the experiments; Analyzed and interpreted the data; Contributed reagents, materials, analysis tools or data.

Magnus Nilsson: Conceived and designed the experiments; Contributed reagents, materials, analysis tools or data.

## Data availability statement

Data will be made available on request.

## Funding

This work was supported by the Lindhagastiftelsen, OM Perssons donationsfond, Stiftelsen Ellen och Henrik Sjöbrings minnesfond, Fonden för psykisk hälsa, and partially by Lund University Faculty of Medicine's Summer Research Scholarship 2022. (The funding sources have no involvement at any stage in the article's creation.)

## Declaration of competing interest

The authors declare that they have no known competing financial interests or personal relationships that could have appeared to influence the work reported in this paper.
