# Supplementary material for: A validation of the Swedish self-concept and Identity Measure (SCIM) and its association with mental health problems
Source: Heliyon. 2023 Jul 11;9(7):e18151. doi: 10.1016/j.heliyon.2023.e18151 (PMC10372226; doi:10.1016/j.heliyon.2023.e18151)
Supplement: Multimedia component 1 [file mmc1.pdf]

1. $Efternamn$, $Förnamn$

Hjälpsökande vid självskadebeteende. Vilken roll spelar attityder, personlighet och exponering för självskadebeteende?
Denna enkät består av 11 sidor med frågor om dig, dina tankar och dina handlingar. Vänligen besvara frågorna genom att kryssa för det alternativ som passar bäst för just dig. Läs frågorna noggrant och se till att svara på samtliga frågor.

Så här fyller du i pappersenkäten
Nedan ser du hur du markerar ett svarsalternativ, och hur du avmarkerar ett redan gjort val.

☒ Korrekt markerat svarsalternativ

☐ Inkorrekt markerat svarsalternativ, krysset ska vara mitt i rutan

☒ Inkorrekt markerat svarsalternativ, krysset är alltför kraftigt

■ Ångrat val, svarsalternativet räknas <u>inte</u> som markerat

Korta frågor om dig

Födelseår

Jag identifierar mig som:

☐ Kvinna
☐ Man
☐ Annan
☐ Vill ej berätta

Under större delen av min uppväxt bodde jag:

☐ På landsbygden
☐ I en mindre ort (t.ex. Åkarp)
☐ I en mellanstor stad (t.ex. Lund)
☐ I en storstad (t.ex. Stockholm)

För närvarande bor jag

☐ På landsbygden
☐ I en mindre ort (t.ex. Åkarp)
☐ I en mellanstor stad (t.ex. Lund)
☐ I en storstad (t.ex. Stockholm)

Jag bor:

☐ Med min partner, sambo eller make/maka.

☐ Ensam (t.ex. i en lägenhet)

☐ Med min ursprungsfamilj (t.ex. med mina föräldrar)

☐ Med andra vuxna (t.ex. i kollektiv, inneboende eller studentkorridor).

☐ Annat, nämligen...

Jag har:

☐ En förälder som är född utomlands.

☐ Två föräldrar som är födda utomlands.

☐ Inga föräldrar som är födda utomlands.

Jag är:

☐ Född i Sverige

☐ Född i ett av de nordiska länderna (Norge, Danmark, Finland eller Island)

☐ Född utanför Norden

Jag är:

☐ Singel

☐ Pojkvän/Flickvän

☐ Gift/Partnerskap

Frågor om självskadebeteende

Jag har eller har haft svårigheter med mitt känslomässiga eller psykiska mående (t.ex. varit deprimerad, upplevt mycket ångest eller dylikt) på ett sådant sätt att jag har haft svårt att hantera dem.

☐ Tidigare i livet men ej under de senaste 6 månaderna.

☐ Ej tidigare i livet men under de senaste 6 månaderna

☐ Både tidigare i livet och under de senaste 6 månaderna

☐ Vare sig tidigare i livet eller under de senaste 6 månaderna

Jag har eller har haft självskadebeteende. Självskadebeteende definieras som att avsiktligt och upprepat skada sig själv men utan avsikten att dö, t.ex. genom att skära, riva, sticka, bränna eller slå sig själv.

- [ ] Tidigare i livet men ej under de senaste 6 månaderna.
- [ ] Ej tidigare i livet men under de 6 månaderna
- [ ] Både tidigare i livet och under de senaste 6 månaderna
- [ ] Vare sig tidigare i livet eller under de senaste 6 månaderna

Hur många gånger har du skadat dig under de senaste 6 månaderna?

- [ ] 0 gånger
- [ ] 1 gång
- [ ] 2 gånger
- [ ] 3 gånger
- [ ] 4 gånger
- [ ] 5 gånger
- [ ] Fler än 5 gånger

Hur många gånger har du skadat dig tidigare i livet innan de senaste 6 månaderna?

- [ ] 0 gånger
- [ ] 1 gång
- [ ] 2 gånger
- [ ] 3 gånger
- [ ] 4 gånger
- [ ] 5 gånger
- [ ] Fler än 5 gånger

Frågor om hjälpsökande för självskadebeteende.

Om jag skulle drabbas av självskadebeteende skulle jag söka hjälp:

- [ ] Ja
- [ ] Troligen ja
- [ ] Troligen nej
- [ ] Nej
- [ ] Vet ej

Om jag skulle söka hjälp för självskadebeteende skulle jag gjort det via följande. Fyll i alla som stämmer.

- ☐ Via vänner och/eller familj
- ☐ Via olika informella forum på internet eller sociala medier.
- ☐ Via skolhälsovård, studenthälsan eller företagshälsovård, t.ex. via skolsköterska
- ☐ Via ett trossamfund, t.ex. Svenska Kyrkan
- ☐ Via frivillig- eller brukarorganisation, t.ex. självmordslinjen
- ☐ Via primärvården, t.ex. vårdcentral.
- ☐ Via privat vårdgivare (t.ex. via privatpraktiserande psykolog)
- ☐ Via psykiatrin
- ☐ Annan, nämligen:…

Jag har bett om eller sökt hjälp för självskadebeteende:

- ☐ Tidigare i livet men ej under de senaste 6 månaderna.
- ☐ Ej tidigare i livet men under de 6 månaderna
- ☐ Både tidigare i livet och under de senaste 6 månaderna
- ☐ Vare sig tidigare i livet eller under de senaste 6 månaderna

Till vem eller vad sökte du hjälp? Fyll i alla som stämmer.

- ☐ Sökte hjälp och stöd hos vänner och/eller familj
- ☐ Sökte hjälp och stöd via olika informella forum på internet eller sociala medier
- ☐ Sökte hjälp via skolhälsovård, studenthälsan eller företagshälsovård, t.ex. via skolsköterska
- ☐ Sökte hjälp via ett trossamfund, t.ex. Svenska Kyrkan
- ☐ Sökte hjälp via frivillig- eller brukarorganisation, t.ex. självmordslinjen
- ☐ Sökte hjälp via primärvården, t.ex. vårdcentral.
- ☐ Sökte hjälp via privat vårdgivare (t.ex. via privatpraktiserande psykolog)
- ☐ Sökte hjälp via psykiatrin
- ☐ Annan, nämligen:

Har någon nu eller tidigare uppmanat dig att söka hjälp för självskadebeteende?

- ☐ Ja
- ☐ Nej

Vem uppmanade dig att söka hjälp? Fyll i alla som stämmer.

☐ Vän(-ner)

☐ Sambo/Partner/maka/make/pojkvän/flickvän

☐ Övrig familj

☐ Skolpersonal

☐ Vårdpersonal

☐ Annan, nämligen...

Jag erhåller eller har erhållit professionell hjälp för självskadebeteende.

☐ Tidigare i livet men ej under de senaste 6 månaderna.

☐ Ej tidigare i livet men under de 6 månaderna

☐ Både tidigare i livet och under de senaste 6 månaderna

☐ Vare sig tidigare i livet eller under de senaste 6 månaderna

Har du en anhörig eller vän som har eller har haft självskadebeteende?

☐ Ja

☐ Nej

Hur har du fått information om hens självskadebeteende?

☐ Personen har själv berättat om sitt självskadebeteende

☐ Fick information om personens självskadebeteende av en annan person än hen själv

☐ Fick information via sociala medier

☐ På annat sätt, nämligen...

När fick du reda på hens självskadebeteende?

☐ Tidigare i livet men ej under de senaste 6 månaderna.

☐ Ej tidigare i livet men under de 6 månaderna

☐ Både tidigare i livet och under de senaste 6 månaderna

Frågor om sociala relationer

	Instämmer inte alls	Instämmer i mycket låg grad	Instämmer i låg grad	Instämmer delvis	Instämmer i hög grad	Instämmer i mycket hög grad	Instämmer helt
Det finns en för mig betydelsefull person i min närhet när jag behöver det.	☐	☐	☐	☐	☐	☐	☐
Det finns en för mig betydelsefull person i min närhet med vilken jag kan dela glädje och sorg.	☐	☐	☐	☐	☐	☐	☐
Min familj försöker verkligen att hjälpa mig.	☐	☐	☐	☐	☐	☐	☐
Jag får den känslomässiga hjälp och det stöd av min familj som jag behöver.	☐	☐	☐	☐	☐	☐	☐
Det finns en för mig betydelsefull person som verkligen bidrar till mitt välmående.	☐	☐	☐	☐	☐	☐	☐
Mina vänner försöker verkligen att hjälpa mig.	☐	☐	☐	☐	☐	☐	☐
Om något går fel kan jag räkna med hjälp från mina vänner.	☐	☐	☐	☐	☐	☐	☐
Jag kan prata om mina problem med min familj.	☐	☐	☐	☐	☐	☐	☐
Jag har vänner med vilka jag kan dela glädje och sorg.	☐	☐	☐	☐	☐	☐	☐
Det finns en för mig betydelsefull person i min närhet som bryr sig om mina känslor.	☐	☐	☐	☐	☐	☐	☐
Min familj är villig att hjälpa mig i mina beslut.	☐	☐	☐	☐	☐	☐	☐
Jag kan prata om mina problem med mina vänner.	☐	☐	☐	☐	☐	☐	☐

Frågor om ditt mående.

Har du under de senaste två veckorna besvärats av något av detta:

	Inte alls	Flera dagar	Mer än hälften av dagarna	Nästan varje dag
Känt mig nervös, orolig, spänd.	☐	☐	☐	☐
Inte kunnat låta bli att ängslas	☐	☐	☐	☐
Ängslats för mycket om olika saker.	☐	☐	☐	☐
Haft svårt att koppla av.	☐	☐	☐	☐
Varit så rastlös att det varit svårt att sitta still	☐	☐	☐	☐
Varit retlig och lättstörd.	☐	☐	☐	☐
Varit rädd, som om något förfärligt skulle hända.	☐	☐	☐	☐

Hur mycket har dessa problem besvärat dig de senaste 7 dagarna?

	Inte alls	Lite grann	Måttligt	Ganska mycket	Väldigt mycket
Känner mig nedstämd.	☐	☐	☐	☐	☐
Känner inget intresse för saker och ting	☐	☐	☐	☐	☐
Känner inget intresse för saker och ting.	☐	☐	☐	☐	☐
Känner mig energifattig eller långsammare än vanligt.	☐	☐	☐	☐	☐
Känns som allting är ansträngande.	☐	☐	☐	☐	☐
Oroar mig alltför mycket för saker och ting.	☐	☐	☐	☐	☐
Detta är en kontrollfråga. Svara "Inte alls".	☐	☐	☐	☐	☐
Klandrar mig själv för saker och ting.	☐	☐	☐	☐	☐

Frågor om attityderHur ställer du dig idag till följande påståenden?

Att personer skadar sig själv är förståeligt.

Människor med självskadebeteende bör inte klandras.

Självskadebeteende är för mig obegripligt.

Att personer skadar sig själv är moraliskt förkastligt.

Jag blir provocerad när människor blir arga på självskadebeteende.

Frågor om problem i vardagenDetta avsnitt handlar om andra aspekter och eventuella problem i din vardag. Tänk på hur det har varit under det senaste året när du besvarar dessa frågor.

Under det senaste året…

	Ja	Nej
Har det varit problem i någon av dina nära relationer på grund av många gräl eller ett flertal uppbrott?	☐	☐
Har du avsiktligt skadat dig själv fysiskt (t.ex. slagit, skurit eller bränt dig själv), eller har du försökt att ta ditt eget liv?	☐	☐
Har du haft minst två andra problem med impulsivitet (t.ex. att du hetsätit, gjort av med för mycket pengar, druckit för mycket, eller fått utbrott)?	☐	☐
Har du haft kraftiga humörsvängningar?	☐	☐
Har du ofta känt dig arg, eller agerat på ett ilsket, sarkastiskt eller spydigt sätt?	☐	☐
Har du ofta känt att du inte kan lita på andra människor?	☐	☐
Har du ofta känt dig overklig, eller som att sakerna omkring dig var overkliga?	☐	☐
Har du känt dig konstant tom inombords?	☐	☐
Har du ofta haft känslan av att du inte vet vem du är, eller att du inte har någon egen identitet?	☐	☐
Har du gjort desperata försök att inte känna dig övergiven eller bli övergiven (t.ex. ringt någon upprepade gånger för att försäkra dig om att han eller hon fortfarande bryr sig, bönat till någon att inte lämna dig, eller klamrat dig fast vid någon fysiskt)?	☐	☐

Frågor om att hantera känslor

Vad god markera hur ofta följande påståenden stämmer för dig.

	Nästan aldrig (0-10% av tiden)	Ibland (11-35% av tiden)	Ca hälften av tiden (35-65% av tiden)	Det mesta av tiden (66-90% av tiden)	Nästan alltid (91-100% av tiden)
Jag har svårt att förstå mig på mina känslor	☐	☐	☐	☐	☐
Jag är förvirrad över hur jag känner mig	☐	☐	☐	☐	☐

	Nästan aldrig (0-10% av tiden)	Ibland (11-35% av tiden)	Ca hälften av tiden (35-65% av tiden)	Det mesta av tiden (66-90% av tiden)	Nästan alltid (91-100% av tiden)
När jag blir känslomässigt upprörd, så... ...har jag svårt att få något arbete gjort	☐	☐	☐	☐	☐
...förlorar jag kontrollen	☐	☐	☐	☐	☐
...tror jag att det kommer att ta lång tid innan det går över	☐	☐	☐	☐	☐
...tror jag att det slutar med att jag blir deprimerad	☐	☐	☐	☐	☐
...har jag svårt att fokusera på andra saker	☐	☐	☐	☐	☐
När jag blir känslomässigt upprörd, så... ...känner jag mig utom kontroll	☐	☐	☐	☐	☐
...skäms jag över mig själv för att jag känner som jag gör	☐	☐	☐	☐	☐
...känns det som att jag är svag	☐	☐	☐	☐	☐
...har jag svårt att kontrollera mitt beteende	☐	☐	☐	☐	☐
...tror jag inte att det finns något jag kan göra för att jag ska må bättre	☐	☐	☐	☐	☐
När jag blir känslomässigt upprörd, så... ...blir jag irriterad på mig själv för att jag känner så	☐	☐	☐	☐	☐
...det här är en kontrollfråga, markera "Det mesta av tiden"	☐	☐	☐	☐	☐
...börjar jag tycka illa om mig själv	☐	☐	☐	☐	☐
...har jag svårt att tänka på något annat	☐	☐	☐	☐	☐
...upplever jag mina känslor som överväldigande	☐	☐	☐	☐	☐

Frågor om dina åsikter kring psykisk vårdFör att besvara följande frågor behövs vissa termer förtydligas. 1. Med mentalvårdspersonal menas t.ex. psykologer, psykiatriker, socialarbetare och kuratorer. 2. Problem med psykisk hälsa inkluderar allt från personliga svårigheter (tex att någon nära dig dör) till psykisk ohälsa (tex ångest, nedstämdhet). Vänligen markera på linjen det som bäst representerar din åsikt. Till exempel, om du tror att det vore ytterst meningslöst att söka hjälp för dina svårigheter, så markerar du längst till vänster under "meningslöst". Om du inte vet svaret eller inte kan bestämma dig för ett svar så väljer du alternativ precis i mitten. Om jag hade svårigheter med psykisk ohälsa så tror jag att söka hjälp från mentalvårdspersonal vore…

Frågor om att hantera felNär saker går fel i vårt liv eller inte fungerar så som vi hoppats, och vi känner att vi kunde ha gjort bättre ifrån oss, kan vi ibland få negativa och självkritiska tankar och känslor. Dessa kan ta sin form i att vi känner oss värdelösa, odugliga eller underlägsna andra. Men människor kan också försöka att vara stödjande gentemot sig själva.

	Inte alls likt mig	Lite likt mig	Ganska likt mig	Mycket likt mig	Precis likt mig
När saker går fel för mig så... ...blir jag lätt besviken på mig själv	☐	☐	☐	☐	☐
...finns det en del av mig som nedvärderar mig själv.	☐	☐	☐	☐	☐
...kan jag påminna mig om positiva saker med mig själv.	☐	☐	☐	☐	☐
...har jag svårt för att hålla min ilska och frustration under kontroll.	☐	☐	☐	☐	☐
...har jag lätt för att förlåta mig själv.	☐	☐	☐	☐	☐
När saker går fel för mig så... ...finns det en del av mig som inte tycker att jag är bra nog.	☐	☐	☐	☐	☐
...känner jag mig ledsen av mina egna självkritiska tankar.	☐	☐	☐	☐	☐
...tycker jag fortfarande om att vara jag.	☐	☐	☐	☐	☐
...har jag blivit så arg på mig själv att jag velat såra eller skada mig själv.	☐	☐	☐	☐	☐
...känner jag äckel gentemot mig själv.	☐	☐	☐	☐	☐
När saker går fel för mig så... ...kan jag ändå känna mig värd att älskas och accepteras.	☐	☐	☐	☐	☐
...slutar jag att bry mig om mig själv.	☐	☐	☐	☐	☐
...har jag lätt för att tycka om mig själv.	☐	☐	☐	☐	☐
...minns jag och ältar över mina misslyckanden.	☐	☐	☐	☐	☐
...hånar jag mig själv.	☐	☐	☐	☐	☐
När saker går fel för mig så... ...är jag snäll och stöttande gentemot mig själv.	☐	☐	☐	☐	☐

	Inte alls likt mig	Lite likt mig	Ganska likt mig	Mycket likt mig	Precis likt mig
...kan jag inte acceptera misslyckanden och bakslag utan attj jag känner mig otillräcklig.	☐	☐	☐	☐	☐
...tycker jag att jag förtjänar min självkritik.	☐	☐	☐	☐	☐
...kan jag bry mig om och ta hand om mig själv.	☐	☐	☐	☐	☐
...detta är en kontrollfråga. Svara "Inte alls likt mig".	☐	☐	☐	☐	☐
När saker går fel för mig så... ...finns det en del av mig som vill bli av med de delar som jag inte gillar hos mig själv.	☐	☐	☐	☐	☐
...uppmuntrar jag mig själv inför framtiden.	☐	☐	☐	☐	☐
...gillar jag inte att vara mig själv.	☐	☐	☐	☐	☐

Frågor om din syn på dig själv

	Håller absolut inte med	Håller inte med	Håller mestad-els inte med	Håller varken med eller inte med	Håller mestad-els med	Håller med	Håller absolut med
Jag vet vad jag tror på och sätter värde på	☐	☐	☐	☐	☐	☐	☐
När någon beskriver mig, vet jag om de har rätt eller fel.	☐	☐	☐	☐	☐	☐	☐
När jag tittar på barndomsbilder känner jag att det finns en röd tråd som förbinder mitt förflutna till den jag är idag.	☐	☐	☐	☐	☐	☐	☐
Ibland väljer jag en person och försöker vara precis som den, även när jag är ensam.	☐	☐	☐	☐	☐	☐	☐
Jag vet vem jag är.	☐	☐	☐	☐	☐	☐	☐
Jag ändras mycket beroende på situationen.	☐	☐	☐	☐	☐	☐	☐
För det mesta gillar jag den jag är.	☐	☐	☐	☐	☐	☐	☐
Jag har aldrig riktigt vetat vad jag tycker och tror.	☐	☐	☐	☐	☐	☐	☐
Jag känner som om att jag är någon annan än mig själv.	☐	☐	☐	☐	☐	☐	☐
Jag känner mig som ett pussel där bitarna inte passar ihop.	☐	☐	☐	☐	☐	☐	☐
Jag är bra.	☐	☐	☐	☐	☐	☐	☐
Jag imiterar andra människor istället för att vara mig själv.	☐	☐	☐	☐	☐	☐	☐
Jag känner mig hel.	☐	☐	☐	☐	☐	☐	☐
Jag har haft samma slags intressen under lång tid.	☐	☐	☐	☐	☐	☐	☐
Jag är så olika med olika människor att jag är osäker på vem jag är ”på riktigt”.	☐	☐	☐	☐	☐	☐	☐
Jag är trasig.	☐	☐	☐	☐	☐	☐	☐

	Håller absolut inte med	Håller inte med	Håller mestad-els inte med	Håller varken med eller inte med	Håller mestad-els med	Håller med	Håller absolut med
När jag minns min barndom känner jag samhörighet med den som jag var då.	☐	☐	☐	☐	☐	☐	☐
Jag känner mig vilse när jag funderar över vem jag är.	☐	☐	☐	☐	☐	☐	☐
Åtminstone en person ser mig som den jag verkligen är.	☐	☐	☐	☐	☐	☐	☐
Jag har alltid en bra uppfattning om vad som är viktigt för mig.	☐	☐	☐	☐	☐	☐	☐
Jag är så lik vissa människor att jag ibland upplever att vi är samma person.	☐	☐	☐	☐	☐	☐	☐
Jag är i grund och botten samma person som jag alltid varit.	☐	☐	☐	☐	☐	☐	☐
Jag känner mig tom inombords, som en person utan själ.	☐	☐	☐	☐	☐	☐	☐
Mina åsikter kan skifta snabbt från en ytterlighet till en annan.	☐	☐	☐	☐	☐	☐	☐
Jag vet inte längre vem jag är.	☐	☐	☐	☐	☐	☐	☐
Jag är mer kapabel när jag är med andra än när jag är själv.	☐	☐	☐	☐	☐	☐	☐
Ingen vet vem jag är egentligen är.	☐	☐	☐	☐	☐	☐	☐
Jag försöker bete mig likadant som de jag är tillsammans med (intressen, musik, klädstil) och det förändras hela tiden.	☐	☐	☐	☐	☐	☐	☐
Jag är komplett bara när jag är med andra människor.	☐	☐	☐	☐	☐	☐	☐

	Håller absolut inte med	Håller inte med	Håller mestadels inte med	Håller varken med eller inte med	Håller mestadels med	Håller med	Håller absolut med
Vad som är viktigast för mig är något som ändrar sig rätt ofta.	☐	☐	☐	☐	☐	☐	☐

Tack för ditt deltagande.OBS: Glöm inte att trycka på "Skicka nu" nedan när du är färdig med enkäten. Om du har självskadebeteende eller mår dåligt psykiskt finns det flera platser du kan söka dig hjälp. Sök i första hand din vårdcentral eller studenthälsan. På 1177 kan du hitta mer information och hur du kontaktar dessa. Följ länkarna nedan: https://www.1177.se/ https://www.lu.se/studera/livet-som-student/service-och-stod/studenthalsan Du kan även kontakta Magnus Nilsson för information om var och hur du kan söka hjälp: E-mail: magnus.nilsson@med.lu.se Telefon: 073-9077304 .
